
JOURNAL INFORMATION
==============================
NLM Title Abbreviation: ScientificWorldJournal
Journal ID: ScientificWorldJournal
NLM Journal ID: 101131163
Journal ID: TSWJ

The Scientific World Journal

ISSN: 2356-6140
EISSN: 1537-744X
Publisher: Wiley

ARTICLE INFORMATION
==============================
PMCID: PMC5956377
PMID: 15523560
DOI: 10.1100/tsw.2004.139
Article ID: 760575
Article version: 1
Subjects: Case Study

An Unusual Cause of Urinary Retention in the Elderly Patient

Hashim Hashim 1 *
Hellawell Giles 2
Kahn Leon 2
1 Bristol Urological Institute, Urology, UK
2 Chase Farm Hospital, Urology, UK
*Hashim Hashim: hashim@doctors.org.ukAcademic Editor: Anthony Atala

Publication date: 2004
Electronic publication date: 2004 Oct 22
Volume: 4
First page: 885
Last page: 886
Received 2004 May 26; Revised 2004 Sep 1; Accepted 2004 Sep 1
Copyright: Copyright © 2004 Hashim Hashim et al.
Copyright year: 2004
License: This is an open access article distributed under the Creative Commons Attribution License, which permits unrestricted use, distribution, and reproduction in any medium, provided the original work is properly cited.
License URL: https://creativecommons.org/licenses/by/3.0/

Keywords: foreign body, acute urinary retention, elderly, rectum

==============================
Case Report
*TheScientificWorldJOURNAL* (2004) 4, 885–886
ISSN 1537-744X; DOI 10.1100/tsw.2004.139

### An Unusual Cause of Urinary Retention in the Elderly Patient

Hashim Hashim[1,*], Giles Hellawell[2], and Leon Kahn[2]
[1]*Bristol Urological Institute, Urology and* [2]*Chase Farm Hospital, Urology*

**E-mail:** hashim@doctors.org.uk

*Received May 26, 2004; Revised September 1, 2004; Accepted September 1, 2004; Published October 22, 2004*

**KEYWORDS**: foreign body, acute urinary retention, elderly, rectum

**DOMAIN:** urology

### INTRODUCTION

Acute urinary retention is a common urological emergency. Benign prostatic enlargement is one of the most common causes. If there is no prostatic enlargement, other causes have to be looked for. We describe an interesting case of urinary retention secondary to the presence of a foreign body in the rectum.

### CASE REPORT

An 83-year-old man with dementia presented with diarrhoea and inability to pass urine. On examination, a bladder was palpable and following catheterization, 600 ml of urine was drained. There was no history of previous urinary or gastrointestinal problems.

A moderately enlarged, benign prostate was found on digital rectal examination (DRE). The patient subsequently developed bleeding in association with the diarrhoea. Examination with a rigid sigmoidoscope was obscured by bleeding, but proctoscopy excluded haemorrhoids as a bleeding source.

Further investigations, including renal function tests, urine microscopy, plain abdominal radiograph and abdominal ultrasound, were normal.

The patient was managed conservatively and commenced on an alpha-blocker. Following removal of the catheter 3 days later, he was unable to pass urine and the catheter was reinserted.

A further rectal bleed led to flexible sigmoidoscopy to determine the source of the bleeding. It was not possible to pass the sigmoidoscope beyond 15 cm and again vision was obscured by bleeding. Following rectal washout, a red foreign body was found to be occluding the rectum.

This was removed manually and was an aerosol cap with a diameter of 5 cm. This retention was probably caused by direct prostatic/urethral impingement with the foreign body that was not felt on DRE and subsequently pushed up by the sigmoidoscope. On direct questioning, the patient denied any knowledge of the foreign body. He made an unremarkable recovery and voided after removal of his catheter and was discharged without complication.

*Corresponding author address: Bristol Urological, Institute,
Southmead Hospital, Westbury-on-Trym, Bristol, BS10 5NB, UK
©2004 with author.

### CONCLUSION

Urinary retention is a common urological emergency accounting for more than 20% of surgical admissions. Placement of a urethral urinary catheter relieves the acute situation allowing further investigation and treatment of contributing causes. Bladder outflow obstruction accounts for the majority of cases in patients over the age of 60, but gastrointestinal[1] and gynaecological[2] causes may also lead to urinary retention.

Many patients are elderly and may have a degree of cognitive impairment. This first report, in the English literature, of a rectal foreign body leading to urinary retention illustrates the need to investigate thoroughly when obvious causes are not present in this patient group.

### REFERENCES

1. Philip, J., Whittestone, T.H., and Hamilton, S.G.I. (2000) An unusual case of acute urinary retention. *Br. J. Urol. Int*. **85(4),** 557.
2. Brown, R.C., Hawtrey, C.E., and Rose, E.F. (1975) Leiomyoma of vesicovaginal septum causing urinary retention. *Urology* **6(3),** 379–381.

**This article should be referenced as follows:**

Hashim, H., Hellawell, G., and Kahn, L. (2004) An unusual cause of urinary retention in the elderly patient. *TheScientificWorldJOURNAL* **4**, 885–886.

**Handling Editor:**

Anthony Atala, Principal Editor for *Urology* — a domain of *TheScientificWorldJOURNAL*.
